# Emergence of Livestock‐Associated Methicillin‐Resistant *Staphylococcus aureus* ST398 in Wild Birds, Brazil

**DOI:** 10.1002/mbo3.70131

**Published:** 2025-11-11

**Authors:** Mateus Rocha Ribas, Felipe Vásquez‐Ponce, Rodrigo Cardoso, Dany Mesa, Gustavo Rocha, Victor Felipe Wolleck, Juliana Lemos Dal Pizzol, Izadora Borgmann Frizzo de Assunção, Vinicius Pais e Oliveira, Gabriel Salvador, Amanda Tfardoski Rodrigues, Gregory Batista Melocco, Fernanda Esposito, Johana Becerra, Nilton Lincopan, Fabienne Antunes Ferreira, Thaís Cristine Marques Sincero, Jussara Kasuko Palmeiro, Sheila Rezler Wosiacki, Silvia Cristina Osaki

**Affiliations:** ^1^ Laboratório de Microbiologia Molecular Aplicada (MiMA), Departamento de Análises Clínicas Centro de Ciências da Saúde, Universidade Federal de Santa Catarina Trindade, Florianópolis Santa Catarina Brazil; ^2^ Departamento de Microbiologia Instituto de Ciências Biomédicas, Universidade de São Paulo, São Paulo São Paulo Brazil; ^3^ 4Biomics Curitiba Paraná Brazil; ^4^ Laboratório de Ecologia de Vertebrados (LabEV), Universidade Federal do Paraná, Setor Palotina Palotina Paraná Brazil; ^5^ Laboratório de Saúde Única (LaSaUn), Universidade Federal do Paraná, Setor Palotina Palotina Paraná Brazil; ^6^ Laboratório de Genética Molecular Bacteriana (GeMBac), Universidade Federal de Santa Catarina Florianópolis Santa Catarina Brazil; ^7^ Programa de Pós‐Graduação em Produção Sustentável e Saúde Animal, Universidade Estadual de Maringá Umuarama Paraná Brazil

**Keywords:** genomic analysis, livestock, methicillin‐resistant *Staphylococcus aureus*, one‐health, resistome, wildlife

## Abstract

Antimicrobial‐resistant and virulent *Staphylococcus aureus* strains are spreading across diverse environments and hosts, but studies on Brazilian wildlife remain limited. From April to December 2021, oropharyngeal swabs were collected from 197 wild birds spanning five orders, 25 families, and 54 species in São Camilo State Park, a protected Atlantic Forest fragment facing significant pressure from surrounding agricultural landscapes. *S. aureus* was detected in 12.2% of the birds, including 27 methicillin‐susceptible *S. aureus* (MSSA) and two Methicillin‐resistant (MRSA) isolates. MSSA strains showed high inducible Macrolide‐Lincosamide‐Streptogramin B (MLSB) resistance, with 37% carrying the *blaZ* gene for penicillin resistance, and antimicrobial‐resistant isolates frequently harboring the *scn* gene. Genomic sequencing identified both MRSA strains as ST398, marking the first report of MRSA ST398 in Brazilian wildlife. These strains displayed a broad resistome, including genes for resistance to multiple antimicrobial classes, as well as a comprehensive virulome, although in vivo assays with *Galleria mellonella* suggested low virulence. Phylogenomic analysis clustered the MRSA strains with MSSA from swine in northeastern Brazil, suggesting that these strains likely originated in livestock, acquired the SCC*mec* element, and spread into natural ecosystems. These findings suggest a possible spillover of livestock‐associated antimicrobial‐resistant *S. aureus* into a protected forest fragment, highlighting the potential for anthropogenic microbial threats to reach wildlife and underscoring the importance of including wild species in antimicrobial resistance surveillance, especially in fragmented ecosystems increasingly exposed to human activities.

## Graphical abstract

In a protected forest fragment in southern Brazil, 197 wild birds were sampled, with 12.2% carrying *Staphylococcus aureus*. Two MRSA ST398 strains, the first reported in Brazilian wild birds, were identified. Genomic analysis revealed resistance and virulence genes, linking these strains to pigs and underscoring the critical need for resistance monitoring in wildlife.

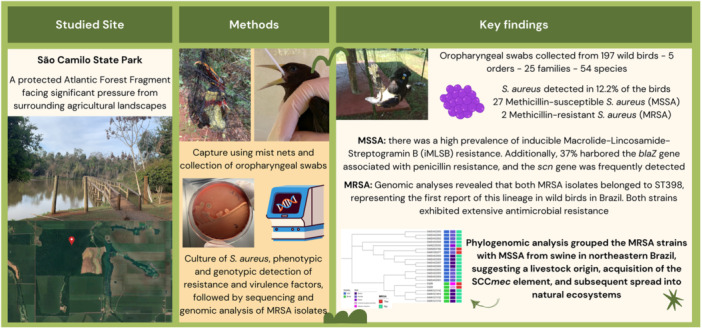

## 1 Introduction

Antimicrobial resistance is one of the greatest threats to global health, with *Staphylococcus aureus* emerging as a key pathogen in this crisis due to its remarkable adaptability and ability to colonize diverse hosts and thrive across diverse environments (Haag et al. 2019; Silva et al. 2020; Howden et al. 2023; Song et al. 2024). This versatility allows it to exist as a commensal organism in the microbiota of humans and animals while also posing significant threats by causing infections ranging from mild to life‐threatening (Krismer et al. 2017; Guo et al. 2020; Cheung et al. 2021). The rise of antimicrobial resistance has propelled *S. aureus* to the forefront of One Health research, particularly regarding methicillin‐resistant *S. aureus* (MRSA), which is resistant to nearly all beta‐lactams and often exhibits high virulence and multidrug resistance (Lee et al. 2018; Algammal et al. 2020).

Classified as a high‐priority pathogen by the World Health Organization (World Health Organization. 2024), MRSA has extended its presence beyond hospital and community settings into livestock and, more recently, into natural ecosystems (Lakhundi and Zhang 2018; Hou et al. 2023). This expansion highlights the growing interconnectedness between human, animal, and environmental health (Denissen et al. 2022). Furthermore, resistant and virulent MRSA strains have been detected in wild animals, including mammals and birds (Heaton et al. 2020; Abdullahi et al. 2021; Martínez‐Seijas et al. 2023), raising concerns about their potential spillover into natural ecosystems and wildlife populations (Dolejská 2020; Ramos et al. 2022; Ramos and Cunha 2024).

Brazil, with its vast territorial expanse, is home to an exceptional diversity of biomes, making it one of the most biodiverse countries on the planet. Among these, the Atlantic Forest stands out as a biodiversity hotspot, rich in species yet severely threatened by urban expansion and the conversion of land into agricultural and livestock areas (Rezende et al. 2018; Grelle et al. 2021; Casallanovo et al. 2024). This transformation of the landscape fosters closer interactions among humans, domestic animals, and wildlife, thereby heightening the risk of pathogen spillover events and accelerating the spread of antimicrobial‐resistant bacteria into natural and protected environments (Faust et al. 2018; Gibb et al. 2020; Wiemeyer et al. 2021; Blanco‐Peña et al. 2024; Ribas et al. 2025).

Birds, as crucial sentinels and reservoirs of antimicrobial‐resistant bacteria (Bonnedahl and Järhult 2014), are especially relevant in this context. However, studies on the microbiota of free‐living wildlife in Brazil, particularly regarding *S. aureus*, remain scarce, limiting the understanding of how environmental changes like forest fragmentation influence pathogen dynamics from a One Health perspective.

This study aims to address this gap by investigating *S. aureus* strains in wild birds inhabiting a protected fragment of the Atlantic Forest, an area under significant agricultural pressure. The research focuses on analyzing their resistance patterns and virulence traits, shedding light on the potential risks these strains pose to the ecosystem and public health. Notably, for the first time, we report MRSA isolates of the international ST398 clone in wild birds in Brazil, highlighting the ecological implications of the dissemination of high‐priority pathogens within Atlantic Forest fragments. Also, the increasing presence of livestock‐associated strains in Brazilian natural environments.

## 2 Materials and Methods

### 2.1 Study Settings and Sample Collection of Wild Birds

**Figure 1:**
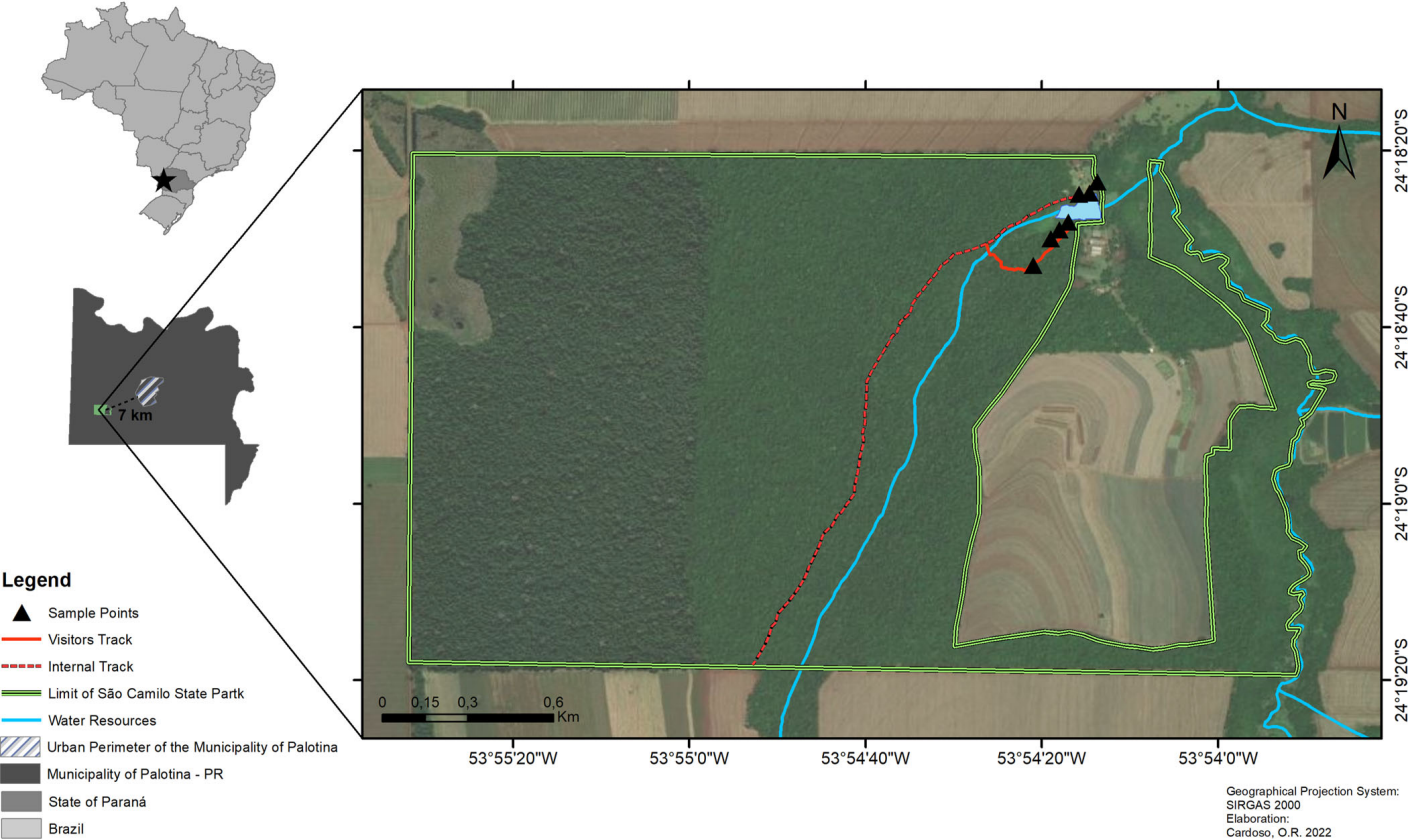
Location of São Camilo State Park, situated in the municipality of Palotina, state of Paraná, Brazil, highlighting the mist nets (black arrows) placed throughout the study.

This study was conducted in São Camilo State Park (hereafter “São Camilo”), a 385.34‐hectare protected area of Seasonal Semideciduous Forest within the Atlantic Forest biome, located in the municipality of Palotina, western Paraná, southern Brazil (24°18′20″ S, 53°54′15″ W) (Figure 1). The park is subject to intense anthropogenic pressure due to surrounding agro‐industrial expansion, leading to significant habitat fragmentation. To investigate the presence of antimicrobial‐resistant *Staphylococcus aureus* in wild bird populations, seven mist nets were installed at distinct locations within the park and operated over a 28‐day period between April and December 2021. Nets were deployed at dawn and remained active for 6 h daily. Captured birds (*n* = 197) across five orders, 25 families, and 54 species (Appendix 1) were carefully removed, placed in cotton fabric holding bags, and transported to a field station for sample collection and species identification. At the station, oropharyngeal swabs were collected from each bird and immediately immersed in Brain Heart Infusion (BHI) broth supplemented with 6.5% NaCl for incubation. Following sampling, all birds were released at their capture sites. The migratory behavior of the sampled bird species was classified following the criteria established by Somenzari et al. (2018).

### 2.2 Culture Processing, Bacterial Identification, and Phenotypic and Genotypic Characterization

Samples were incubated at 37°C for 18 to 24 h and then cultured on 5% sheep blood agar plates. Isolate identification was conducted using Matrix‐Assisted Laser Desorption/Ionization Time‐of‐Flight (MALDI‐TOF) mass spectrometry (Bruker MALDI Biotyper). Antimicrobial susceptibility testing was performed by disk‐diffusion method against the following antimicrobial agents (in µg/disk): penicillin G (10 U), cefoxitin (30), clindamycin (2), erythromycin (15), gentamicin (10), amikacin (30), tobramycin (10), ciprofloxacin (5), levofloxacin (5), norfloxacin (10), tetracycline (30), tigecycline (15) and trimethoprim/sulfamethoxazole (1.25/23.75). The double‐disk diffusion method with clindamycin and erythromycin disks was performed to determine Macrolide‐Lincosamide‐Streptogramin B (MLSB) resistance phenotypes. Brazilian Committee on Antimicrobial Susceptibility Testing—BrCAST 2025 guidelines were followed, except for testing with penicillin G, which followed the Clinical and Laboratory Standards Institute—CLSI. 2025 standards. The reference strains *S. aureus* ATCC 25923 and *S. aureus* ATCC 29213 were used as quality control strains. On the other hand, the antimicrobial resistance genes *mecA*, *blaZ*, *ermA*, *ermB*, *ermC*, *ermT*, and *aacA*‐*aphD*, along with virulence genes *sea*, *seg*, *seh*, *sen*, *tst*, *lukED*, *lukFS*, *icaAD*, *icaBC*, *sasG*, *fnbpA*, *fnbpB*, and *scn*, were selected for genotypic characterization of the isolates by polymerase chain reaction (PCR). Multiplex PCR was conducted to determine the SCC*mec* type in methicillin‐resistant isolates. To control for DNA extraction quality, a partial region of the 16S rDNA gene was also amplified by PCR. The used primers and amplification conditions are detailed in Appendix 2.

### 2.3 In Vivo Virulence Assays in the *Galleria mellonella* Infection Model

To evaluate the virulence potential of MRSA strains, an in vivo experiment was carried out with the *Galleria mellonella* infection model (Tsai et al. 2016; Sheehan et al. 2019). Groups of 10 larvae (230–280 mg) were inoculated with 10 μL of 2 × 10^6^ CFU/larvae of each bacterial strain in PBS. A control group was injected with 10 μL PBS to rule out death from physical trauma. *S. aureus* AD7a587 was used as a virulent control, while *S. aureus* ATCC 25923 served as a non‐virulent control. After treatment, larvae were incubated at 37°C, and survival was monitored hourly for 120 h. Two biological replicates were done per strain. Survival curves were plotted with the Kaplan‐Meier method, and the log‐rank test (*p* < 0.05) was used for statistical analysis (Origin Software, USA).

### 2.4 Whole Genome Sequence Analysis

The genome sequencing of the samples confirmed that MRSA was performed. For the whole genome sequence, genomic DNA was extracted using the PureLink™ Genomic DNA Mini Kit (Thermo Fisher Scientific, USA). DNA quality and concentration were assessed by NanoDrop spectrophotometer (Thermo Scientific) and Qubit 2.0 fluorometer (Life Technologies, Carlsbad, CA), respectively. The library was constructed using the Nextera DNA Prep kit (Illumina, San Diego, CA, USA) and sequenced using the NextSeq. 550 paired reads platform (2 × 75 bp). Reads were trimmed with Trimmomatic and assembled using CLC Genomics Workbench 12.0.3. The read quality was verified using FastQC (Andrews 2010) version 0.12.1. These reads were subjected to the genomic assembly process using SPAdes (Bankevich et al. 2012) version 3.15.5 (options –cov‐cutoff 30 and –phred‐offset 33). Contigs smaller than 500 base pairs were filtered out. For the identification of virulence and resistance genes in MRSA strains, the ABRicate program (Torsten, n.d.) was used. Within ABRicate, the genomes were analyzed using the following databases: NCBI AMRFinderPlus (Feldgarden et al. 2019), CARD (Jia et al. 2016), ARG‐ANNOT (Gupta et al. 2014), Resfinder (Zankari et al. 2012), MEGARES 2.0 (Doster et al. 2020), PlasmidFinder (Carattoli et al. 2014), and VFDB (Chen et al. 2016). To identify *spa* tandem repeats, the *spa* region was analyzed online using *spa*Typer (http://spatyper.fortinbras.us/), and results were cross‐verified with the Ridom *spa* Server (https://www.spaserver.ridom.de/) for accurate classification. For SCC*mec* detection, *in silico* PCR was performed with Geneious Prime (2024.0.4; Biomatters), both to confirm conventional PCR findings and to screen for a broader range of SCC*mec* types (I to XI). This analysis used gene targets proposed by Yamaguchi et al. (2020).

### 2.5 Phylogenetic Analysis

For phylogenetic analysis based on the core‐genome, 1661 strains of *Staphylococcus aureus* belonging to ST398 circulating at the human‐animal‐environment interface were obtained from NCBI and Pathogenwatch (Argimón et al. 2021) (https://pathogen.watch/genomes/all?genusId=1279&mlst=398&speciesId=1280). All genomes used (1663) were annotated using the Prokka program (Seemann 2014) version 1.14.6, and the annotation files in GFF format were submitted to the Panaroo program (Tonkin‐Hill et al. 2020) using MAFFT (Katoh and Standley 2013) as the aligner (options ‐a core ‐‐aligner mafft) to obtain the already aligned core‐genome (file in ALN extension). Once the aligned core‐genome was obtained, the file was submitted to the IQ‐TREE program (von Haeseler et al. 2014) using the ultrafast bootstrap method (Hoang et al. 2018) with 1000 replicates and the Tamura‐Nei model (Tamura and Nei 1993) (options ‐B 1000 ‐m TN93) to generate the phylogenetic tree of all analyzed strains. Once the preceding phylogenetic relationship has been established, a comparative cgMLST analysis of the 21 closest genomes from *S. aureus* circulating at the human–animal interface was constructed using a concatenated filtered core‐gene alignment from 2335 genes (2,060,121 bp in length) obtained with Panaroo software (https://github.com/gtonkinhill/panaroo), and FastTree 2.1.11. The SNP‐sites software (https://github.com/sanger-pathogens/snp-sites) was used to obtain Single Nucleotide Polymorphisms (SNPs), and the snp‐dists 0.8.2 software (https://github.com/tseemann/snp-dists) was used to build the SNP matrix. Tree visualization, country, host, MRSA. Antimicrobial susceptibility and heatmap of antibiotic resistome were performed using iTol v.6 (https://itol.embl.de/).

### 2.6 Genomic Sequencing Repository

All assembled genomes of the MRSA strains from this study have been deposited in NCBI with the following identifiers: JBAGCW000000000 for the strain hosted by *Cyanoloxia glaucocaerulea* (isolate 271 ‐ SQ688) and JBAGCX000000000 for the strain hosted by *Manacus manacus* (isolate 275 ‐ SQ696).

## 3 Results

### 3.1 Characterization of Methicillin‐Susceptible *Staphylococcus aureus*

**Figure 2:**
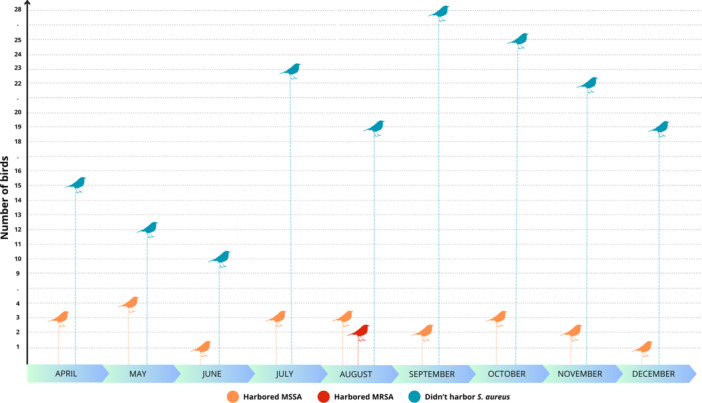
Monthly sampling of wild birds in São Camilo State Park, southern Brazil (April–December 2021), focusing on the temporal distribution of *Staphylococcus aureus* isolates detected in the sampled birds.

*S. aureus* was detected in 12.2% of the sampled birds, resulting in 29 isolates from 24 individuals across four orders (Passeriformes, Columbiformes, Piciformes, and Cuculiformes), 15 families, and 20 species. Of these, 17 species exhibit non‐migratory behavior (i.e., are resident), while only three are known to display partial migratory patterns (Table 1; Appendix 1). The isolates were recovered in every month of the study (Figure 2). Twenty‐seven isolates were identified as Methicillin‐Susceptible *Staphylococcus aureus* (MSSA). From those, approximately 70% (*n* = 19) exhibited inducible MLSB resistance, although none harbored the evaluated *erm* genes. Around 37% (*n* = 10) of the MSSA isolates carried the *blaZ* gene, conferring resistance to penicillin, and 11% (*n* = 3) of these isolates showed resistance to at least one tested aminoglycoside, despite the absence of the *aacA‐aphD* gene. Seven isolates were susceptible to all tested antimicrobial agents (Table 1).

**Table 1:** Antimicrobial resistance and virulence profiles of methicillin‐susceptible *Staphylococcus aureus* isolates recovered from wild birds in São Camilo State Park, southern Brazil.

ID	Specie	Order	Family	Isolate	Resistance phenotype	Resistance genes	Virulence genes
14	*Sicalis flaveola*	Passeriformes	Thraupidae	46	Susceptible	(‐)	*icaAD ‐ icaBC ‐ sasG ‐ fnbA ‐ lukDE*
15	*Cyanocorax chrysops*	Passeriformes	Corvidae	48	CLI ‐ ERI ‐ GEN ‐ AMI ‐ TOB	(‐)	*icaAD ‐ icaBC ‐ fnbA ‐ scn*
16	*Setophaga pitiayumi*	Passeriformes	Parulidae	53	CLI ‐ ERI	(‐)	*icaAD ‐ icaBC ‐ fnbA ‐ scn*
				54	CLI ‐ ERI	(‐)	*icaAD ‐ icaBC ‐ fnbA ‐ scn*
29	*Pipra fasciicauda*	Passeriformes	Pipridae	107	PEN ‐ CLI ‐ ERI ‐ TOB	*bla*Z	*icaAD ‐ icaBC ‐ fnbA ‐ scn*
30	*Dacnis cayana*	Passeriformes	Thraupidae	108	PEN ‐ CLI ‐ ERI	*bla*Z	*icaAD ‐ icaBC ‐ fnbA ‐ scn*
31	*Basileuterus culicivorus*	Passeriformes	Parulidae	109	CLI ‐ ERI	(‐)	*icaAD ‐ icaBC ‐ fnbA ‐ scn*
32	*Basileuterus culicivorus*	Passeriformes	Parulidae	111	PEN ‐ CLI ‐ ERI	*bla*Z	*icaAD ‐ icaBC ‐ fnbA ‐ scn*
41	*Arremon polionotus*	Passeriformes	Passerellidae	135	PEN ‐ CLI ‐ ERI	*bla*Z	*icaAD ‐ icaBC ‐ fnbA ‐ scn*
55	*Basileuterus culicivorus*	Passeriformes	Parulidae	172	PEN ‐ CLI ‐ ERI	*bla*Z	*icaAD ‐ icaBC ‐ fnbA ‐ scn*
62	*Leptotila verreauxi*	Columbiformes	Columbidae	191	Susceptible	(‐)	*icaAD ‐ icaBC ‐ fnbA*
				192	Susceptible	(‐)	*icaAD ‐ icaBC ‐ fnbA ‐ scn ‐ lukDE*
67	*Crotophaga ani*	Cuculiformes	Cuculidae	208	Susceptible	(‐)	*icaAD ‐ icaBC ‐ fnbA*
72	*Sicalis flaveola*	Passeriformes	Thraupidae	221	PEN ‐ CLI ‐ ERI	*bla*Z	*icaAD ‐ icaBC ‐ fnbA ‐ scn*
75	*Dryocopus lineatus*	Piciformes	Picidae	229	PEN ‐ CLI ‐ ERI	*bla*Z	*icaAD ‐ icaBC ‐ fnbA ‐ scn*
79	*Turdus amaurochalinus*	Passeriformes	Turdidae	240	AMI ‐ TOB	(‐)	*icaAD ‐ icaBC ‐ sasG ‐ fnbA ‐ lukDE*
120	*Pipra fasciicauda*	Passeriformes	Pipridae	324	Susceptible	(‐)	*icaAD ‐ icaBC ‐ sasG ‐ fnbA ‐ lukDE*
				333	Susceptible	(‐)	*icaAD ‐ icaBC ‐ sasG ‐ fnbA ‐ lukDE*
121	*Thamnophilus doliatus*	Passeriformes	Thamnophilidae	325	CLI ‐ ERI	(‐)	*icaAD ‐ icaBC ‐ fnbA ‐ scn*
				326	CLI ‐ ERI	(‐)	*icaAD ‐ icaBC ‐ fnbA ‐ scn*
138	*Thraupis sayaca*	Passeriformes	Thraupidae	364	CLI ‐ ERI	(‐)	*icaAD ‐ icaBC ‐ fnbA ‐ scn*
151	*Columbina talpacoti*	Columbiformes	Columbidae	395	PEN ‐ CLI ‐ ERI	*bla*Z	*icaAD ‐ icaBC ‐ fnbA ‐ scn*
153	*Pachyramphus validus*	Passeriformes	Tityridae	399	PEN ‐ CLI ‐ ERI	*bla*Z	*icaAD ‐ icaBC ‐ fnbA ‐ scn*
162	*Cacicus haemorrhous*	Passeriformes	Icteridae	425	CLI ‐ ERI	(‐)	*icaAD ‐ icaBC ‐ fnbA ‐ scn*
				426	CLI ‐ ERI	(‐)	*icaAD ‐ icaBC ‐ fnbA ‐ scn*
176	*Cnemotriccus fuscatus*	Passeriformes	Tyrannidae	462	PEN ‐ CLI ‐ ERI	*bla*Z	*icaAD ‐ icaBC ‐ fnbA ‐ scn*
191	*Leptopogon amaurocephalus*	Passeriformes	Rhynchocyclidae	496	Susceptible	(‐)	*icaAD ‐ icaBC ‐ sasG ‐ fnbA ‐ seh ‐ lukDE*

Abbreviations: AMI, amikacin; CLI, clindamycin; ERI, erythromycin; GEN, gentamicin; PEN, penicillin; TOB, tobramycin.

Regarding the virulence gene profile, six strains carried the *lukED* gene, and one isolate of these isolates also harbored the *seh* enterotoxin gene. The *scn* gene was present in 74% (*n* = 20) of MSSA isolates. Notably, only one isolate carried both *lukED* and *scn* genes. The chromosomal intercellular adhesion gene cluster *icaABCD*, which is associated with biofilm production, was found in all isolates. Additionally, the fibronectin‐binding protein A gene was present in all isolates, while the fibronectin‐binding protein B gene was absent. The surface‐anchored protein G gene was identified in five isolates (Table 1).

### 3.2 Characterization of Methicillin‐Resistant *Staphylococcus aureus*

Two MRSA isolates were found in *Cyanoloxia glaucocaerulea* and *Manacus manacus*, both exhibiting a broad resistance phenotype. The MRSA isolate from *C. glaucocaerulea* was resistant to penicillin, cefoxitin, ciprofloxacin, norfloxacin, levofloxacin, gentamicin, tobramycin, tetracycline, and trimethoprim/sulfamethoxazole, along with constitutive MLSB resistance. Similarly, the isolate from *M. manacus* showed resistance to penicillin, cefoxitin, ciprofloxacin, levofloxacin, gentamicin, tobramycin, tetracycline, and trimethoprim/sulfamethoxazole, and exhibited constitutive MLSB resistance to clindamycin and erythromycin. Due to the high‐priority status of MRSA as a pathogen and its broad antimicrobial resistance, both isolates were selected for comprehensive genome analysis.

### 3.3 Genomic and Phylogenetic Characterization of MRSA Isolates

Genomic analysis identified both MRSA isolates as belonging to sequence type (ST) 398, a lineage often associated with livestock worldwide. The isolate from *C. glaucocaerulea* was classified as *spa* type t1456 (tandem repeat sequence: 08‐16‐02‐25), while the isolate from *M. manacus* was classified as *spa* type t1451 (tandem repeat sequence: 08‐16‐02‐25‐34‐25). The SCC*mec* element in both isolates was confirmed as type V via conventional and *in silico* PCR.

An extensive resistome was found in both MRSA strains, with resistance genes identified for multiple antimicrobial classes. Detected genes included those conferring resistance to β‐lactams (*mecA*, *blaZ*), aminoglycosides [*ant(6)‐Ia*, *aac(6′)‐aph(2″)*, *spw*, *ant(9)*, *aph‐Stph*, *aac(3)*], macrolides (*ermT*), phenicols (*fexA*), lincosamides (*isaE*, *inuB*), tetracycline (*tetM*, *tetL*), and trimethoprim (*dfrG*). Chromosomal point mutations were also observed in *gyrA*, *grlA*, and *grlB*, conferring resistance to quinolones. Additionally, several efflux pump genes were identified [*arlR, arlS*, *mgrA*, *norA*, *norB*, *lmrS*, *tet(38)*], further contributing to the multidrug resistance profile of these strains.

**Figure 3:**
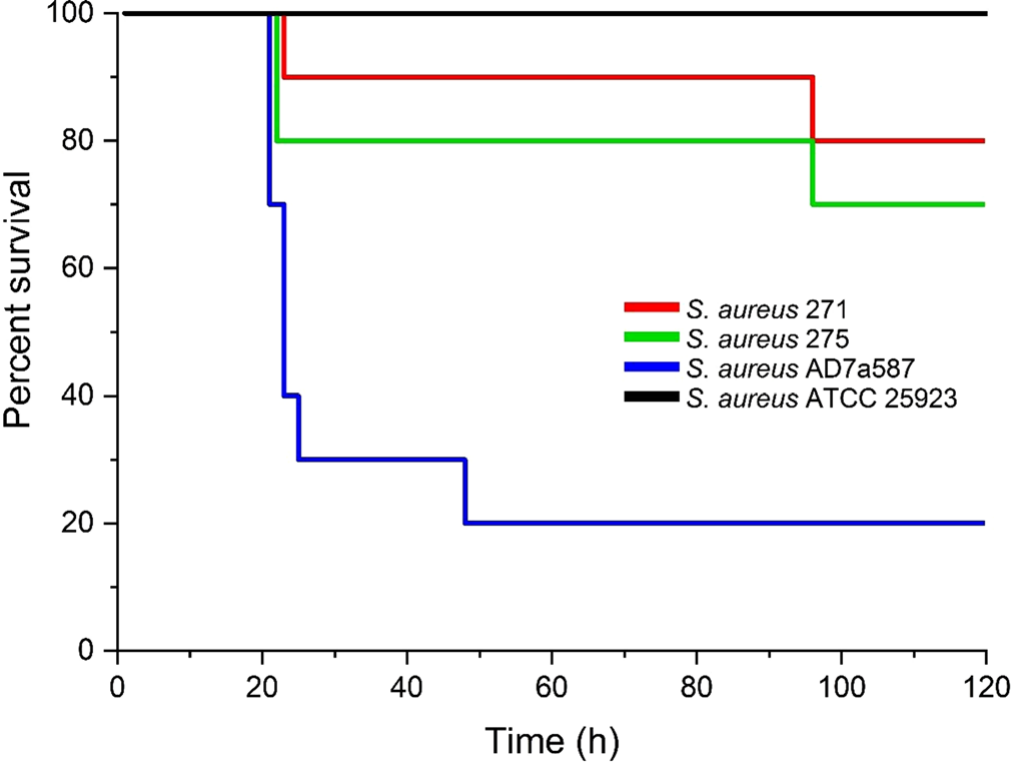
Kaplan–Meier survival plots of *Galleria mellonella* larvae infected with 2 × 106 UFC/larvae of *S. aureus* MRSA 271 and 275 strains. Plots show an average of three independent experiments with 10 larvae per group with mortality monitored daily for 120 h. The clinical strain *S. aureus* AD7a587 was used as a virulent control, and *S. aureus* ATC + C 25923 as a non‐virulent control.

The virulome of both strains included genes involved in adherence (e.g., *sdrC*, *sdrE*, *clfA*, *clfB*), biofilm formation (*ica* operon), immune evasion (capsule synthesis genes *cap8A*‐cap8P), and toxin production (e.g., *hlgA*, *hlgB*, *hlgC*, *hla*, *hld*, *hlb*). These virulence genes are part of the typical accessory genome of *S. aureus* and are associated with colonization and infection capacity (Table 2). The virulence potential of MRSA strains was assessed using a *Galleria mellonella* killing assay. Despite an extensive virulome, both strains exhibited lower mortality rates compared to the virulent strain AD7a587, which is associated with human clinical infections. Specifically, the tested strains caused 20% and 30% mortality within 120 h, while strain AD7a587 resulted in 80% mortality over the same period (Figure 3).

**Table 2:** Genomic and epidemiological characteristics of Methicillin‐Resistant *Staphylococcus aureus* ST398 isolates recovered from wild birds in São Camilo State Park, southern Brazil.

Isolate Information	*Staphylococcus aureus* (Isolate 271 ‐ SQ688)	*Staphylococcus aureus* (isolate 275 ‐ SQ696)
Host	*Cyanoloxia glaucocaerulea* (d'Orbigny & Lafresnaye, 1837)	*Manacus manacus* (Linnaeus, 1766)
Taxonomic Group	Order: Passeriformes ‐ Family: Cardinalidae	Order: Passeriformes ‐ Family: Pipridae
Source	Oropharynx	Oropharynx
Antibiotic Resistance Phenotype	PEN ‐ FOX ‐ CLI ‐ ERY ‐ CIP ‐ NOR ‐ LEV ‐ GEN ‐ TOB ‐ TET ‐ SUL	PEN ‐ FOX ‐ CLI ‐ ERY ‐ CIP ‐ LEV ‐ GEN ‐ TOB ‐ TET ‐ SUL
**Genome information**		
Genome size (bp)	2,761,481	2,761,987
Contig number	61	61
CDSs	2,508	2,510
N. rRNAs	1	1
N. tRNAs	14	14
% GC	32.7%	32.7%
**Epidemiological data**		
MLST	ST398	ST398
SCC*mec*	SCC*mec* type V	SCC*mec* type V
*spa* type	t1456	t1451
**Resistome**		
Aminoglycosides	*ant(6)‐Ia, aac(6′)‐aph(2″), spw, ant(9)*	*ant(6)‐Ia, aac(6′)‐aph(2″), spw, aph‐Stph, aac(3), ant(9)*
β‐lactams	*mecA, blaZ, blaR1, blaI*	*mecA, blaZ, BlaR1, blaI*
Macrolides	*erm(T)*	*erm(T)*
Fluoroquinolones	*gyrA, grlA, grlB* (Mutation)	*gyrA, grlA, grlB* (Mutation)
Phenicols	*fexA*	*fexA*
Lincosamides	*lsa(E), lnu(B), erm(T)*	*lsa(E), lnu(B), erm(T)*
Tetracycline	*tet(M), tet(L)*	*tet(M), tet(L)*
Trimethoprim	*dfrG*	*dfrG*
Efflux pumps	*arlR, arlS, mgrA, lmrS, norA, norB, tet(38)*	*arlR, arlS, mgrA, norA, norB*, *lmrS, tet(38)*
**Virulome**		
Exoenzymes	*aur*	*aur*
Toxins	*hlgA, hlgB, hlgC, hly/hla, hld, hlb, sel26*	*hlgA, hlgB, hlgC, hly/hla, hld, hlb, sel26*
Clumping factor	*clfA, clfB*	*clfA, clfB*
Hemoglobin receptor	*isdA, isdB, isdC, isdD, isdE, isdF, isdG*	*isdA, isdB, isdC, isdD, isdE, isdF, isdG*
Adhesin (PIA) synthesis	*icaA, icaB, icaC, icaD, icaR*	*icaA, icaB, icaC, icaD, icaR*
Serine‐aspartate repeat Sdr proteins	*sdrC, sdrE*	*sdrC, sdrE*
Capsular polysaccharide synthesis	*cap8A, cap8B, cap8C, cap8D, cap8E, cap8F, cap8G, cap8L, cap8M, cap8N, cap8O, cap8P*	*cap8A, cap8B, cap8C, cap8D, cap8E, cap8F, cap8G, cap8L, cap8M, cap8N, cap8O, cap8P*
Type VII secretion system	*esxA, esaA, essA, esaB, essB*	*esxA, esaA, essA, esaB, essB*
others	*sbi, spa, ebp, hysA, strB, adsA, lip, coa, map, sspA, sspB, sspC, geh*	*sbi, spa, ebp, hysA, strB, adsA, lip, coa, map, sspA, sspB, sspC, geh*

Abbreviations: CIP, ciprofloxacin; CLI, clindamycin; ERI, erythromycin; FOX, cefoxitin; GEN, gentamicin; LEV, levofloxacin; NOR, norfloxacin; PEN, Penicillin; SUL, sulfamethoxazole/Trimethoprim; TET, tetracycline; TOB, tobramycin.

**Figure 4:**
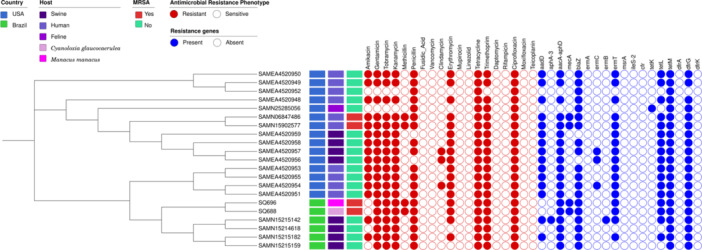
Phylogenetic tree of 21 *Staphylococcus aureus* ST398 isolates, showing their genomic relatedness, host species, country of origin, methicillin resistance (MRSA), antimicrobial resistance profiles, and resistance genes. Colored boxes indicate the host (e.g., swine, human, bird) and the country (Brazil or USA) where each strain was isolated. Red and green squares show MRSA and MSSA status, respectively. The heatmap displays phenotypic resistance to 19 antibiotics (red = resistant; white = sensitive) and the presence of 18 resistance genes (blue = present; white = absent), allowing comparison between resistance profiles and genetic similarity among isolates.

The MRSA isolates from São Camilo's wild birds displayed a close genetic relationship with MSSA strains from swine in Paraíba, northeastern Brazil, and with previously reported *S. aureus* ST398 strains from swine, humans, and felines in the United States. The phylogenetic analysis identified between 0 and 166 single‐nucleotide polymorphism (SNP) differences among the strains; notably, the São Camilo isolates from wild birds differed by only 27 SNPs. Furthermore, fewer than 80 SNPs separated these bird isolates from the Brazilian swine‐associated MSSA strains. These findings suggest potential cross‐species transmission and underscore the genetic proximity between wild bird and livestock‐associated strains (Figure 4, Appendix 3).

## 4 Discussion

São Camilo, a 385.34‐hectare remnant of Seasonal Semideciduous Forest within the Atlantic Forest biome, holds exceptional ecological value as one of the few preserved forest fragments in western Paraná. Its high bird biodiversity, comprising over 220 species, underscores its role as a reservoir of complex ecological interactions and a potential interface for pathogen exchange between wildlife, livestock, and the environment (Rocha Ribas et al. 2023). However, like many remnants of the Atlantic Forest, São Camilo faces increasing pressure from surrounding agro‐industrial activities (Kramer et al. 2023). The proximity to intensive livestock farming and large‐scale monocultures likely contributes to environmental contamination with resistant bacteria and antimicrobial residues, creating selective pressures that may facilitate the emergence or persistence of resistant strains in local fauna. The detection of both MRSA and MSSA in wild birds from this fragment reinforces the role of avifauna as sentinels for environmental dissemination of *Staphylococcus aureus*.

This scenario highlights the urgent need to better understand the dynamics of antimicrobial resistance (AMR) in wild bird populations, particularly within ecologically sensitive and human‐impacted regions such as São Camilo. A more comprehensive understanding of AMR in *Staphylococcus* from wild birds in Brazil is hindered by the scarcity of epidemiological and molecular data. However, key studies have identified the presence of resistant *Staphylococcus* spp. in diverse contexts, including illegally trafficked birds (Matias et al. 2018), urban environments (Ewbank et al. 2021; Ribas et al. 2025), and even within protected conservation areas (Saraiva et al. 2021; Ribas et al. 2025). These findings, although fragmented, point to a broader and potentially silent dissemination of resistance that may be going largely undetected. Our study builds on this emerging body of work by incorporating both phenotypic and genomic data from wild birds sampled in a natural forest fragment, thereby contributing new insights into the environmental spread of antimicrobial resistance in Brazil.

In this study, *S. aureus* was isolated from 12.2% of sampled wild birds, a prevalence comparable to the global average of 10.3% reported by Abdullahi et al. (2021). Moreover, *S. aureus* was identified in 37% of the sampled bird species (20 species across different orders and families), suggesting substantial host diversity and ecological plasticity of this bacterium in wild bird populations. Although sampling was conducted over nonconsecutive days spanning multiple months, *S. aureus* was detected in every sampling period, suggesting that the presence of this bacterium is not restricted to a specific season. This temporal consistency strengthens the reliability of our prevalence estimates. Nevertheless, we recognize that certain ecological or microhabitat‐level factors, such as bird movement patterns or local environmental conditions, could influence detection rates and merit further investigation in future studies.

Interestingly, most birds harboring *S. aureus* in our study were non‐migratory species, which reinforces the hypothesis that antimicrobial‐resistant strains, or those carrying virulence factors of anthropogenic origin, were likely acquired through local or regional exposure. This finding corroborates that resident birds may act as sentinels of environmental contamination in natural and protected areas influenced by human activity (Ribas et al. 2025). Although they do not undertake long‐distance migrations, these species often move across fragmented landscapes, such as forest edges, agroecosystems, and peri‐urban zones, potentially facilitating short‐range dissemination of resistant bacteria. In this context, a variety of environmental reservoirs and transmission vectors, such as synanthropic rodents (Devanathan et al. 2024), marsupials (Santana et al. 2022), nonhuman primates (Sales et al. 2024), water sources (Tsai et al. 2020), insects (Gwenzi et al. 2021), livestock (Ramos and Cunha 2024), and human visitors (Chong et al. 2020), may contribute to the circulation of *S. aureus* in these ecosystems. Despite this complexity, comprehensive environmental surveillance studies remain scarce in Brazil, particularly those assessing multiple environmental matrices. This gap highlights the urgent need for integrated One Health approaches to better elucidate the ecological dynamics of *S. aureus* transmission in protected and natural environments.

These ecological dynamics, shaped by the limited mobility of resident bird species and the influence of surrounding anthropogenic pressures, may not only facilitate the transmission of *S. aureus* across environmental interfaces but also create selective conditions that favor the emergence and persistence of resistant phenotypes. In this context, among MSSA isolates, antimicrobial susceptibility testing revealed a high prevalence of inducible MLSB phenotype. Although resistance to clindamycin and erythromycin, often linked to the *erm* gene family, has been documented in *S. aureus* from wildlife (Wardyn et al. 2012; Ruiz‐Ripa et al. 2019), the elevated occurrence of this phenotype in our study is noteworthy. Interestingly, common resistance determinants such as *ermA*, *ermC*, and *ermT* were not detected, suggesting the involvement of alternative resistance mechanisms, less common *erm* variants, or even mutations in these genes that may have hindered PCR detection. In this context, further genomic investigation will be essential to elucidate the underlying resistance determinants.

β‐lactamase production mediated by the *blaZ* gene was identified in 41% (*n* = 12) of *S. aureus* isolates, surpassing the 28% reported in *Staphylococcus* spp. from trafficked birds in Rio de Janeiro (Matias et al. 2018). These enzymes, commonly detected in clinical and livestock isolates (Neelam et al. 2022; Di Gregorio et al. 2023), are increasingly reported in wildlife (Gómez et al. 2015; Martínez‐Seijas et al. 2023). Their detection in a natural forest remnant emphasizes the environmental penetration of resistance traits typically associated with anthropized ecosystems.

Another significant finding was the detection of the *scn* gene in all MSSA isolates exhibiting the iMLSB phenotype and penicillinase production. *scn* encodes the Staphylococcal Complement Inhibitor (SCIN), a component of the immune evasion cluster (IEC) characteristic of human‐adapted *S. aureus* (De Jong et al. 2018). Its presence in wildlife suggests possible zooanthroponotic transmission, echoing similar concerns raised by other authors (Abdullahi et al. 2021; Gómez et al. 2021).

The oropharyngeal MRSA carriage rate in birds (1%) was slightly lower than global reports (3.4%; Abdullahi et al. 2021). Notably, MRSA was identified in *Manacus manacus* (non‐migratory, frugivorous) and *Cyanoloxia glaucocaerulea* (partially migratory, omnivorous) (Wilman et al. 2014; Somenzari et al. 2018), expanding the known host range of MRSA‐ST398 in neotropical avifauna.

Genomic analyses revealed that both strains belonged to sequence type ST398, a lineage historically associated with livestock, particularly swine (Zarazaga et al. 2018; Silva et al. 2023). ST398 is recognized for its ecological versatility and capacity to colonize multiple host species across environmental interfaces, representing one of the *S. aureus* lineages with the greatest zoonotic potential (Graveland et al. 2011; Haag et al. 2019). The resistome of these isolates included the *ermT* gene (conferring cMLSB resistance) and the *tetM* gene (linked to Tn916), while lacking key IEC genes such as *scn*, *sea*, *sep*, and *chp*, a genomic profile commonly reported in livestock‐associated strains (Price et al. 2012).

Phylogenetic analysis revealed close relatedness to MSSA strains isolated from swine in northeastern Brazil (Santos et al. 2021), raising the hypothesis that the MRSA isolates detected in birds may have originated from livestock‐associated MSSA strains, which later acquired SCC*mec* elements, possibly under antimicrobial pressure. Although the genomic data, strain characteristics, and local livestock production (Machado et al. 2025) strongly suggest a link between the isolates from wild birds and livestock‐associated lineages, the directionality of this transmission remains uncertain, and alternative pathways cannot be excluded. In this context, further genomic and metagenomic studies, including environmental samples, livestock (especially swine), humans, and other wild animals, will be essential to clarify the evolutionary trajectory and transmission dynamics.

Their subsequent detection in wild birds may reflect a dynamic interface between agriculture and wildlife, underscoring the environmental dissemination of resistance. To our knowledge, this represents the first report of MRSA‐ST398 in wildlife in Brazil. Until recently, ST398 was rarely reported in the country, with sporadic detection in livestock and occasional human infections (Silva et al. 2014; Lima et al. 2017). However, its increasing presence in urban and clinical settings, including individuals with no livestock exposure (André‐Neto et al. 2017), suggests wider dissemination. In wildlife, a study involving neotropical primates also reported MSSA isolates belonging to this clonal complex, highlighting its dissemination in Atlantic Forest fragments near urbanized areas (Sales et al. 2024). Recent genomic studies have confirmed clonal complex 398 (CC398) as the dominant MSSA lineage in Brazil (Di Gregorio et al. 2023). Alarmingly, these strains have shown the capacity to acquire SCC*mec* and virulence genes, especially during periods of heightened antimicrobial use, such as the COVID‐19 pandemic.

Although this study focuses on a single forest fragment, it reveals broader implications for other Atlantic Forest remnants under similar anthropogenic pressure. Hundreds of forest patches in Brazil may be facing comparable microbial threats yet remain unexplored. The co‐circulation of virulent MSSA and adaptive MRSA strains with zoonotic potential in wildlife within a protected area raises significant concerns for public and environmental health. These findings underscore the urgency for expanded One Health surveillance strategies to monitor antimicrobial resistance at the human‐animal‐environment interface.

## 5 Conclusion

This study highlights the emergence of antimicrobial‐resistant *S. aureus*, including livestock‐associated MRSA‐ST398, in wild birds from a protected Atlantic Forest fragment. The detection of resistance and virulence traits in a protected area under pressure points to the silent spread of anthropogenic microbial threats across ecological boundaries, a phenomenon that may be even more pronounced in smaller, unprotected forest fragments, an all‐too‐common scenario across multiple biomes in Brazil. These findings stress the urgent need to incorporate wildlife into antimicrobial resistance surveillance frameworks, particularly in biodiversity hotspots increasingly influenced by human activity.

## Author Contributions

**Mateus Rocha Ribas:** conceptualization, data curation; investigation; methodology; visualization; writing – original draft; writing – review and editing. **Felipe Vásquez‐Ponce:** data curation; formal analysis; software; investigation; writing – review and editing. **Rodrigo Cardoso:** data curation; formal analysis; software. **Dany Mesa:** data curation; formal analysis; software. **Gustavo Rocha:** investigation; data curation; writing – review and editing. **Victor Felipe Wolleck:** investigation; data curation; writing – review & editing**. Juliana Lemos Dal Pizzol:** investigation; data curation; writing – review and editing. **Izadora Borgmann Frizzo de Assunção:** investigation; visualization; data curation; writing – review and editing. **Vinícius Pais e Oliveira:** investigation; data curation. **Gabriel Salvador:** investigation; data curation. **Amanda Tfardoski Rodrigues:** investigation; data curation. **Gregory Batista Melocco:** data curation; formal analysis; software. **Fernanda Esposito:** data curation; formal analysis; software. **Johana Becerra:** investigation; data curation; formal analysis. **Fabienne Antunes Ferreira:** resources, validation. **Nilton Lincopan:** resources, validation. **Thaís Cristine Marques Sincero:** resources, supervision; validation. **Jussara Kasuko Palmeiro:** resources, validation; visualization; project administration; supervision; funding acquisition; writing – review and editing. **Sheila Rezler Wosiacki:** supervision; validation. **Silvia Cristina Osaki (in memoriam):** conceptualization; investigation; funding acquisition; methodology; resources, validation; visualization; project administration; supervision.

## Ethics Statement

This study was authorized by the *Sistema de Autorização e Informação em Biodiversidade* (SISBIO) under License #75086‐3 and approved by the *Comissão de Ética no Uso de Animais—UFPR—Setor Palotina* (CEUA/Palotina—Protocol No. 09/2020). The captures in São Camilo State Park were authorized by the state environmental agency *Instituto Água e Terra* (Protocol No. 17.317.251‐6).

## Conflicts of Interest

The authors declare no conflicts of interest.

## Data Availability

All assembled genomes of the MRSA strains from this study have been deposited in NCBI with the following identifiers: JBAGCW000000000 for the strain hosted by *Cyanoloxia glaucocaerulea* (isolate 271 ‐ SQ688) and JBAGCX000000000 for the strain hosted by *Manacus manacus* (isolate 275 ‐ SQ696). The data that support the findings of this study are openly available in NCBI at https://www.ncbi.nlm.nih.gov/datasets/genome/, reference number JBAGCW000000000 and JBAGCX000000000.

## Appendix 1 Inventory of Wild Birds Documented Throughout the Study

**Table:**

Individual	Month/2021	Species	Order	Family	Migratory Patterns	*Staphyloccocus aureus*
1	April	*Columbina talpacoti* (Temminck, 1811)	Columbiformes	Columbidae	Resident	(‐)
2	April	*Megaceryle torquata* (Linnaeus, 1766)	Coraciiformes	Alcedinidae	Resident	(‐)
3	April	*Sicalis flaveola* (Linnaeus, 1766)	Passeriformes	Thraupidae	Resident	(‐)
4	April	*Thamnophilus doliatus* (Linnaeus, 1764)	Passeriformes	Thamnophilidae	Resident	(‐)
5	April	*Crotophaga ani* Linnaeus, 1758	Cuculiformes	Cuculidae	Resident	(‐)
6	April	*Crotophaga ani* Linnaeus, 1758	Cuculiformes	Cuculidae	Resident	(‐)
7	April	*Basileuterus culicivorus* (Deppe, 1830)	Passeriformes	Parulidae	Resident	(‐)
8	April	*Sicalis flaveola* (Linnaeus, 1766)	Passeriformes	Thraupidae	Resident	(‐)
9	April	*Dacnis cayana* (Linnaeus, 1766)	Passeriformes	Thraupidae	Resident	(‐)
10	April	*Passer domestiscus* (Linnaeus, 1758)	Passeriformes	Passeridae	Resident	(‐)
11	April	*Columbina talpacoti* (Temminck, 1811)	Columbiformes	Columbidae	Resident	(‐)
12	April	*Columbina talpacoti* (Temminck, 1811)	Columbiformes	Columbidae	Resident	(‐)
13	April	*Basileuterus culicivorus* (Deppe, 1830)	Passeriformes	Parulidae	Resident	(‐)
14	April	*Sicalis flaveola* (Linnaeus, 1766)	Passeriformes	Thraupidae	Resident	Present
15	April	*Cyanocorax chrysops* (Vieillot, 1818)	Passeriformes	Corvidae	Resident	Present
16	April	*Setophaga pitiayumi* (Vieillot, 1817)	Passeriformes	Parulidae	Resident	Present
17	April	*Manacus manacus* (Linnaeus, 1766)	Passeriformes	Pipridae	Resident	(‐)
18	April	*Columbina talpacoti* (Temminck, 1811)	Columbiformes	Columbidae	Resident	(‐)
19	May	*Columbina talpacoti* (Temminck, 1811)	Columbiformes	Columbidae	Resident	(‐)
20	May	*Todirostrum cinereum* (Linnaeus, 1766)	Passeriformes	Rhynchocyclidae	Resident	(‐)
21	May	*Sicalis flaveola* (Linnaeus, 1766)	Passeriformes	Thraupidae	Resident	(‐)
22	May	*Furnarius rufus* (Gmelin, 1788)	Passeriformes	Furnariidae	Resident	(‐)
23	May	*Arremon polionotus Bonaparte, 1850*	Passeriformes	Passerellidae	Resident	(‐)
24	May	*Turdus leucomelas* Vieillot, 1818	Passeriformes	Turdidae	Resident	(‐)
25	May	*Basileuterus culicivorus* (Deppe, 1830)	Passeriformes	Parulidae	Resident	(‐)
26	May	*Leptopogon amaurocephalus* Tschudi, 1846	Passeriformes	Rhynchocyclidae	Resident	(‐)
27	May	*Columbina talpacoti* (Temminck, 1811)	Columbiformes	Columbidae	Resident	(‐)
28	May	*Sicalis flaveola* (Linnaeus, 1766)	Passeriformes	Thraupidae	Resident	(‐)
29	May	*Pipra fasciicauda* Hellmayr, 1906	Passeriformes	Pipridae	Resident	Present
30	May	*Dacnis cayana* (Linnaeus, 1766)	Passeriformes	Thraupidae	Resident	Present
31	May	*Basileuterus culicivorus* (Deppe, 1830)	Passeriformes	Parulidae	Resident	Present
32	May	*Basileuterus culicivorus* (Deppe, 1830)	Passeriformes	Parulidae	Resident	Present
33	May	*Columbina talpacoti* (Temminck, 1811)	Columbiformes	Columbidae	Resident	(‐)
34	May	*Arremon polionotus Bonaparte, 1850*	Passeriformes	Passerellidae	Resident	(‐)
35	June	*Basileuterus culicivorus* (Deppe, 1830)	Passeriformes	Parulidae	Resident	(‐)
36	June	*Sicalis flaveola* (Linnaeus, 1766)	Passeriformes	Thraupidae	Resident	(‐)
37	June	*Pitangus sulphuratus* (Linnaeus, 1766)	Passeriformes	Tyrannidae	Partially Migratory	(‐)
38	June	*Troglodytes musculus* Naumann, 1823	Passeriformes	Troglodytidae	Resident	(‐)
39	June	*Dendrocolaptes platyrostris* Spix, 1825	Passeriformes	Dendrocolaptidae	Resident	(‐)
40	June	*Picumnus temminckii* Lafresnaye, 1845	Piciformes	Picidae	Resident	(‐)
41	June	*Arremon polionotus Bonaparte, 1850*	Passeriformes	Passerellidae	Resident	Present
42	June	*Columbina talpacoti* (Temminck, 1811)	Columbiformes	Columbidae	Resident	(‐)
43	June	*Pteroglossus castanotis* Gould, 1834	Piciformes	Ramphastidae	Resident	(‐)
44	June	*Pteroglossus castanotis* Gould, 1834	Piciformes	Ramphastidae	Resident	(‐)
45	June	*Cyclarhis gujanensis* (Gmelin, 1789)	Passeriformes	Vireonidae	Resident	(‐)
46	July	*Passer domestiscus* (Linnaeus, 1758)	Passeriformes	Passeridae	Resident	(‐)
47	July	*Hemitriccus margaritaceiventer* (d'Orbigny & Lafresnaye, 1837)	Passeriformes	Rhynchocyclidae	Resident	(‐)
48	July	*Columbina talpacoti* (Temminck, 1811)	Columbiformes	Columbidae	Resident	(‐)
49	July	*Passer domestiscus* (Linnaeus, 1758)	Passeriformes	Passeridae	Resident	(‐)
50	July	*Columbina talpacoti* (Temminck, 1811)	Columbiformes	Columbidae	Resident	(‐)
51	July	*Dysithamnus mentalis* (Temminck, 1823)	Passeriformes	Thamnophilidae	Resident	(‐)
52	July	*Columbina talpacoti* (Temminck, 1811)	Columbiformes	Columbidae	Resident	(‐)
53	July	*Columbina talpacoti* (Temminck, 1811)	Columbiformes	Columbidae	Resident	(‐)
54	July	*Manacus manacus* (Linnaeus, 1766)	Passeriformes	Pipridae	Resident	(‐)
55	July	*Basileuterus culicivorus* (Deppe, 1830)	Passeriformes	Parulidae	Resident	Present
56	July	*Myiarchus ferox* (Gmelin, 1789)	Passeriformes	Tyrannidae	Resident	(‐)
57	July	*Myiarchus ferox* (Gmelin, 1789)	Passeriformes	Tyrannidae	Resident	(‐)
58	July	*Sicalis flaveola* (Linnaeus, 1766)	Passeriformes	Thraupidae	Resident	(‐)
59	July	*Sicalis flaveola* (Linnaeus, 1766)	Passeriformes	Thraupidae	Resident	(‐)
60	July	*Troglodytes musculus* Naumann, 1823	Passeriformes	Troglodytidae	Resident	(‐)
61	July	*Cacicus haemorrhous* (Linnaeus, 1766)	Passeriformes	Icteridae	Resident	(‐)
62	July	*Leptotila verreauxi* Bonaparte, 1855	Columbiformes	Columbidae	Resident	Present
63	July	*Columbina talpacoti* (Temminck, 1811)	Columbiformes	Columbidae	Resident	(‐)
64	July	*Turdus leucomelas* Vieillot, 1818	Passeriformes	Turdidae	Resident	(‐)
65	July	*Sicalis flaveola* (Linnaeus, 1766)	Passeriformes	Thraupidae	Resident	(‐)
66	July	*Sicalis flaveola* (Linnaeus, 1766)	Passeriformes	Thraupidae	Resident	(‐)
67	July	*Crotophaga ani* Linnaeus, 1758	Cuculiformes	Cuculidae	Resident	Present
68	July	*Crotophaga ani* Linnaeus, 1758	Cuculiformes	Cuculidae	Resident	(‐)
69	July	*Columbina talpacoti* (Temminck, 1811)	Columbiformes	Columbidae	Resident	(‐)
70	July	*Zenaida auriculata* (Des Murs, 1847)	Columbiformes	Columbidae	Resident	(‐)
71	July	*Pitangus sulphuratus* (Linnaeus, 1766)	Passeriformes	Tyrannidae	Partially Migratory	(‐)
72	August	*Sicalis flaveola* (Linnaeus, 1766)	Passeriformes	Thraupidae	Resident	Present
73	August	*Platyrinchus mystaceus* Vieillot, 1818	Passeriformes	Platyrinchidae	Resident	(‐)
74	August	*Leptotila verreauxi* Bonaparte, 1855	Columbiformes	Columbidae	Resident	(‐)
75	August	*Dryocopus lineatus* (Linnaeus, 1766)	Piciformes	Picidae	Resident	Present
76	August	*Sicalis flaveola* (Linnaeus, 1766)	Passeriformes	Thraupidae	Resident	(‐)
77	August	*Columbina talpacoti* (Temminck, 1811)	Columbiformes	Columbidae	Resident	(‐)
78	August	*Myiarchus ferox* (Gmelin, 1789)	Passeriformes	Tyrannidae	Resident	(‐)
79	August	*Turdus amaurochalinus* Cabanis, 1850	Passeriformes	Turdidae	Partially Migratory	Present
80	August	*Turdus leucomelas* Vieillot, 1818	Passeriformes	Turdidae	Resident	(‐)
81	August	*Euphonia chlorotica* (Linnaeus, 1766)	Passeriformes	Fringillidae	Resident	(‐)
82	August	*Sicalis flaveola* (Linnaeus, 1766)	Passeriformes	Thraupidae	Resident	(‐)
83	August	*Picumnus temminckii* Lafresnaye, 1845	Piciformes	Picidae	Resident	(‐)
84	August	*Passer domestiscus* (Linnaeus, 1758)	Passeriformes	Passeridae	Resident	(‐)
85	August	*Troglodytes musculus* Naumann, 1823	Passeriformes	Troglodytidae	Resident	(‐)
86	August	*Tachyphonus coronatus* (Vieillot, 1822)	Passeriformes	Thraupidae	Resident	(‐)
87	August	*Turdus amaurochalinus* Cabanis, 1850	Passeriformes	Turdidae	Partially Migratory	(‐)
88	August	*Passer domestiscus* (Linnaeus, 1758)	Passeriformes	Passeridae	Resident	(‐)
89	August	*Passer domestiscus* (Linnaeus, 1758)	Passeriformes	Passeridae	Resident	(‐)
90	August	*Manacus manacus* (Linnaeus, 1766)	Passeriformes	Pipridae	Resident	(‐)
91	August	*Cyanoloxia glaucocaerulea* (d'Orbigny & Lafresnaye, 1837)	Passeriformes	Cardinalidae	Partially Migratory	Present (MRSA)
92	August	*Todirostrum cinereum* (Linnaeus, 1766)	Passeriformes	Rhynchocyclidae	Resident	(‐)
93	August	*Manacus manacus* (Linnaeus, 1766)	Passeriformes	Pipridae	Resident	Present (MRSA)
94	August	*Passer domestiscus* (Linnaeus, 1758)	Passeriformes	Passeridae	Resident	(‐)
95	August	*Sicalis flaveola* (Linnaeus, 1766)	Passeriformes	Thraupidae	Resident	(‐)
96	September	*Cacicus haemorrhous* (Linnaeus, 1766)	Passeriformes	Icteridae	Resident	(‐)
97	September	*Turdus amaurochalinus* Cabanis, 1850	Passeriformes	Turdidae	Partially Migratory	(‐)
98	September	*Cacicus haemorrhous* (Linnaeus, 1766)	Passeriformes	Icteridae	Resident	(‐)
99	September	*Cacicus haemorrhous* (Linnaeus, 1766)	Passeriformes	Icteridae	Resident	(‐)
100	September	*Cacicus haemorrhous* (Linnaeus, 1766)	Passeriformes	Icteridae	Resident	(‐)
101	September	*Cacicus haemorrhous* (Linnaeus, 1766)	Passeriformes	Icteridae	Resident	(‐)
102	September	*Conirostrum speciosum* (Temminck, 1824)	Passeriformes	Thraupidae	Resident	(‐)
103	September	*Columbina talpacoti* (Temminck, 1811)	Columbiformes	Columbidae	Resident	(‐)
104	September	*Leptotila verreauxi* Bonaparte, 1855	Columbiformes	Columbidae	Resident	(‐)
105	September	*Cacicus haemorrhous* (Linnaeus, 1766)	Passeriformes	Icteridae	Resident	(‐)
106	September	*Leptotila verreauxi* Bonaparte, 1855	Columbiformes	Columbidae	Resident	(‐)
107	September	*Turdus leucomelas* Vieillot, 1818	Passeriformes	Turdidae	Resident	(‐)
108	September	*Cacicus haemorrhous* (Linnaeus, 1766)	Passeriformes	Icteridae	Resident	(‐)
109	September	*Molothrus bonariensis* (Gmelin, 1789)	Passeriformes	Icteridae	Resident	(‐)
110	September	*Cacicus haemorrhous* (Linnaeus, 1766)	Passeriformes	Icteridae	Resident	(‐)
111	September	*Turdus amaurochalinus* Cabanis, 1850	Passeriformes	Turdidae	Partially Migratory	(‐)
112	September	*Cacicus haemorrhous* (Linnaeus, 1766)	Passeriformes	Icteridae	Resident	(‐)
113	September	*Cnemotriccus fuscatus* (Wied, 1831)	Passeriformes	Tyrannidae	Resident	(‐)
114	September	*Camptostoma obsoletum* (Temminck, 1824)	Passeriformes	Tyrannidae	Resident	(‐)
115	September	*Pitangus sulphuratus* (Linnaeus, 1766)	Passeriformes	Tyrannidae	Partially Migratory	(‐)
116	September	*Cacicus haemorrhous* (Linnaeus, 1766)	Passeriformes	Icteridae	Resident	(‐)
117	September	*Conirostrum speciosum* (Temminck, 1824)	Passeriformes	Thraupidae	Resident	(‐)
118	September	*Cacicus haemorrhous* (Linnaeus, 1766)	Passeriformes	Icteridae	Resident	(‐)
119	September	*Cacicus haemorrhous* (Linnaeus, 1766)	Passeriformes	Icteridae	Resident	(‐)
120	September	*Pipra fasciicauda* Hellmayr, 1906	Passeriformes	Pipridae	Resident	Present
121	September	*Thamnophilus doliatus* (Linnaeus, 1764)	Passeriformes	Thamnophilidae	Resident	Present
122	September	*Cacicus haemorrhous* (Linnaeus, 1766)	Passeriformes	Icteridae	Resident	(‐)
123	September	*Thraupis sayaca* (Linnaeus, 1766)	Passeriformes	Thraupidae	Resident	(‐)
124	September	*Saltator similis* d'Orbigny & Lafresnaye, 1837	Passeriformes	Thraupidae	Resident	(‐)
125	September	*Elaenia sp*.	Passeriformes	Tyrannidae	*	(‐)
126	October	*Euphonia chlorotica* (Linnaeus, 1766)	Passeriformes	Fringillidae	Resident	(‐)
127	October	*Turdus leucomelas* Vieillot, 1818	Passeriformes	Turdidae	Resident	(‐)
128	October	*Cacicus haemorrhous* (Linnaeus, 1766)	Passeriformes	Icteridae	Resident	(‐)
129	October	*Pachyramphus validus* (Lichtenstein, 1823)	Passeriformes	Tityridae	Partially Migratory	(‐)
130	October	*Turdus leucomelas* Vieillot, 1818	Passeriformes	Turdidae	Resident	(‐)
131	October	*Stelgidopteryx ruficollis* (Vieillot, 1817)	Passeriformes	Hirundinidae	Partially Migratory	(‐)
132	October	*Elaenia sp*.	Passeriformes	Tyrannidae	*	(‐)
133	October	*Columbina talpacoti* (Temminck, 1811)	Columbiformes	Columbidae	Resident	(‐)
134	October	*Tachyphonus coronatus* (Vieillot, 1822)	Passeriformes	Thraupidae	Resident	(‐)
135	October	*Pachyramphus validus* (Lichtenstein, 1823)	Passeriformes	Tityridae	Partially Migratory	(‐)
136	October	*Tyrannus melancholicus* Vieillot, 1819	Passeriformes	Tyrannidae	Partially Migratory	(‐)
137	October	*Euphonia violacea* (Linnaeus, 1758)	Passeriformes	Fringillidae	Resident	(‐)
138	October	*Thraupis sayaca* (Linnaeus, 1766)	Passeriformes	Thraupidae	Resident	Present
139	October	*Myiarchus ferox* (Gmelin, 1789)	Passeriformes	Tyrannidae	Resident	(‐)
140	October	*Leptopogon amaurocephalus* Tschudi, 1846	Passeriformes	Tyrannidae	Resident	(‐)
141	October	*Thraupis sayaca* (Linnaeus, 1766)	Passeriformes	Thraupidae	Resident	(‐)
142	October	*Myiarchus ferox* (Gmelin, 1789)	Passeriformes	Tyrannidae	Resident	(‐)
143	October	*Thamnophilus doliatus* (Linnaeus, 1764)	Passeriformes	Thamnophilidae	Resident	(‐)
144	October	*Tyrannus melancholicus* Vieillot, 1819	Passeriformes	Tyrannidae	Partially Migratory	(‐)
145	October	*Leptotila verreauxi* Bonaparte, 1855	Columbiformes	Columbidae	Resident	(‐)
146	October	*Myiozetetes similis* (Spix, 1825)	Passeriformes	Tyrannidae	Resident	(‐)
147	October	*Elaenia sp*.	Passeriformes	Tyrannidae	*	(‐)
148	October	*Sicalis flaveola* (Linnaeus, 1766)	Passeriformes	Thraupidae	Resident	(‐)
149	October	*Myiarchus ferox* (Gmelin, 1789)	Passeriformes	Tyrannidae	Resident	(‐)
150	October	*Myiarchus ferox* (Gmelin, 1789)	Passeriformes	Tyrannidae	Resident	(‐)
151	October	*Columbina talpacoti* (Temminck, 1811)	Columbiformes	Columbidae	Resident	Present
152	October	*Cacicus haemorrhous* (Linnaeus, 1766)	Passeriformes	Icteridae	Resident	(‐)
153	October	*Pachyramphus validus* (Lichtenstein, 1823)	Passeriformes	Tityridae	Partially Migratory	Present
154	November	*Cacicus haemorrhous* (Linnaeus, 1766)	Passeriformes	Icteridae	Resident	(‐)
155	November	*Cacicus haemorrhous* (Linnaeus, 1766)	Passeriformes	Icteridae	Resident	(‐)
156	November	*Cacicus haemorrhous* (Linnaeus, 1766)	Passeriformes	Icteridae	Resident	(‐)
157	November	*Tachyphonus coronatus* (Vieillot, 1822)	Passeriformes	Thraupidae	Resident	(‐)
158	November	*Sicalis flaveola* (Linnaeus, 1766)	Passeriformes	Thraupidae	Resident	(‐)
159	November	*Cacicus haemorrhous* (Linnaeus, 1766)	Passeriformes	Icteridae	Resident	(‐)
160	November	*Leptopogon amaurocephalus* Tschudi, 1846	Passeriformes	Rhynchocyclidae	Resident	(‐)
161	November	*Myiophobus fasciatus* (Statius Muller, 1776)	Passeriformes	Tyrannidae	Partially Migratory	(‐)
162	November	*Cacicus haemorrhous* (Linnaeus, 1766)	Passeriformes	Icteridae	Resident	Present
163	November	*Euphonia violacea* (Linnaeus, 1758)	Passeriformes	Fringillidae	Resident	(‐)
164	November	*Myiopagis viridicata* (Vieillot, 1817)	Passeriformes	Tyrannidae	Partially Migratory	(‐)
165	November	*Cnemotriccus fuscatus* (Wied, 1831)	Passeriformes	Tyrannidae	Resident	(‐)
166	November	*Manacus manacus* (Linnaeus, 1766)	Passeriformes	Pipridae	Resident	(‐)
167	November	*Myiarchus ferox (Gmelin, 1789)*	Passeriformes	Tyrannidae	Resident	(‐)
168	November	*Dacnis cayana* (Linnaeus, 1766)	Passeriformes	Thraupidae	Resident	(‐)
169	November	*Celeus flavescens* (Gmelin, 1788)	Piciformes	Picidae	Resident	(‐)
170	November	*Dysithamnus mentalis* (Temminck, 1823)	Passeriformes	Thamnophilidae	Resident	(‐)
171	November	*Cacicus haemorrhous* (Linnaeus, 1766)	Passeriformes	Icteridae	Resident	(‐)
172	November	*Trichothraupis melanops* (Vieillot, 1818)	Passeriformes	Thraupidae	Resident	(‐)
173	November	*Sicalis flaveola* (Linnaeus, 1766)	Passeriformes	Thraupidae	Resident	(‐)
174	November	*Turdus leucomelas* Vieillot, 1818	Passeriformes	Turdidae	Resident	(‐)
175	November	*Leptopogon amaurocephalus* Tschudi, 1846	Passeriformes	Rhynchocyclidae	Resident	(‐)
176	November	*Cnemotriccus fuscatus* (Wied, 1831)	Passeriformes	Tyrannidae	Resident	Present
177	November	*Columbina talpacoti* (Temminck, 1811)	Columbiformes	Columbidae	Resident	(‐)
178	December	*Turdus leucomelas* Vieillot, 1818	Passeriformes	Turdidae	Resident	(‐)
179	December	*Sicalis flaveola* (Linnaeus, 1766)	Passeriformes	Thraupidae	Resident	(‐)
180	December	*Sporophila caerulescens* (Vieillot, 1823)	Passeriformes	Thraupidae	Partially Migratory	(‐)
181	December	*Cacicus haemorrhous* (Linnaeus, 1766)	Passeriformes	Icteridae	Resident	(‐)
182	December	*Columbina talpacoti* (Temminck, 1811)	Columbiformes	Columbidae	Resident	(‐)
183	December	*Veniliornis spilogaster* (Wagler, 1827)	Piciformes	Picidae	Resident	(‐)
184	December	*Tachyphonus coronatus* (Vieillot, 1822)	Passeriformes	Thraupidae	Resident	(‐)
185	December	*Arremon polionotus Bonaparte, 1850*	Passeriformes	Passerellidae	Resident	(‐)
186	December	*Manacus manacus* (Linnaeus, 1766)	Passeriformes	Pipridae	Resident	(‐)
187	December	*Cnemotriccus fuscatus (Wied, 1831)*	Passeriformes	Tyrannidae	Resident	(‐)
188	December	*Furnarius rufus* (Gmelin, 1788)	Passeriformes	Furnariidae	Resident	(‐)
189	December	*Elaenia sp*.	Passeriformes	Tyrannidae	*	(‐)
190	December	*Euscarthmus meloryphus* Wied, 1831	Passeriformes	Tyrannidae	Resident	(‐)
191	December	*Leptopogon amaurocephalus* Tschudi, 1846	Passeriformes	Rhynchocyclidae	Resident	Present
192	December	*Elaenia sp*.	Passeriformes	Tyrannidae	*	(‐)
193	December	*Pipra fasciicauda* Hellmayr, 1906	Passeriformes	Pipridae	Resident	(‐)
194	December	*Basileuterus culicivorus* (Deppe, 1830)	Passeriformes	Parulidae	Resident	(‐)
195	December	*Cacicus haemorrhous* (Linnaeus, 1766)	Passeriformes	Icteridae	Resident	(‐)
196	December	*Columbina talpacoti* (Temminck, 1811)	Columbiformes	Columbidae	Resident	(‐)
197	December	*Cacicus haemorrhous* (Linnaeus, 1766)	Passeriformes	Icteridae	Resident	(‐)

* Species with challenging identification. Migratory habits vary between species.

## Appendix 2 Primers and Conditions Used for PCR Amplification

**Table:**

Target	Oligonucleotide Sequence (5′‐3′)	PCR cycles	Amplicon size (bp)	Reference
*16 s rDNA*	F‐GTGCCAGCMGCCGCGGTAA	35 cycles (95°C ‐ 30 s; 56°C ‐ 30 s; 72°C ‐ 45 s)	977	Tanner et al. (2000)
	R‐GGYTACCTTGTTACGACTT			
*icaAD*	F‐TGGCTACTGGGATACTGATA	35 cycles (95°C ‐ 30 s; 55°C ‐ 30 s; 72°C ‐ 45 s)	520	Tang et al. (2012)
	R‐TGGAAATGCGACAAGAACTA			
*icaBC*	F‐ GCCTATCCTTATGGCTTGA	35 cycles (95°C ‐ 30 s; 55°C ‐ 30 s; 72°C ‐ 45 s)	182	
	R‐TGGAATCCGTCCCATCTC			
*sasG*	F‐TATCAACACTTCCGTAACCTTC	35 cycles (95°C ‐ 30 s; 59°C ‐ 30 s; 72°C ‐ 45 s)	159	
	R‐CGTCAGTCACTCATAACGCAGA			
*fnbA*	F‐TCCGCCGAACAACATACC	35 cycles (95°C ‐ 30 s; 54°C ‐ 30 s; 72°C ‐ 45 s)	952	
	R‐TCAAGCACAAGGACCAAT			
*fnbB*	F‐TCTGCGTTATGAGGATTT	35 cycles (95°C ‐ 30 s; 54°C ‐ 30 s; 72°C ‐ 45 s)	452	
	R‐ACAGTAGAGGAAAGTGGG			
*scn*	F‐AGCACAAGCTTGCCAACATCG	35 cycles (94°C ‐ 30 s; 49°C ‐ 30 s; 72°C ‐ 45 s)	258	Van Wamel et al. (2006)
	R‐TTAATATTTACTTTTTAGTGC			
*sea*	F‐GACTTGTATGGTGCTTATTATG	35 cycles (95°C ‐ 30 s; 55°C ‐ 30 s; 72°C ‐ 45 s)	361	Ribeiro et al. (2007)
	R‐AATACTGTCCTTGAGCACCA			
*seg*	F‐AATTATGTGAATGCTCAACCCG	35 cycles (95°C ‐ 30 s; 53°C ‐ 30 s; 72°C ‐ 45 s)	634	
	R‐TATGGAACAAAAGGTACTAGTTC			
*seh*	F‐TCACATCATATGCGAAAGCAG	35 cycles (95°C ‐ 30 s; 55°C ‐ 30 s; 72°C ‐ 45 s)	371	
	R‐CATCTACCCAAACATTAGCAC			
*sen*	F‐TAATGCTGAAGTAGACAAAAAAG	35 cycles (95°C ‐ 30 s; 55°C ‐ 30 s; 72°C ‐ 45 s)	615	
	R‐ AAACTCTGCTCCCACTGAAC			
*tst*	F‐ATCGTAAGCCCTTTGTTG	35 cycles (95°C ‐ 30 s; 55°C ‐ 30 s; 72°C ‐ 45 s)	334	Lindsay et al. (1998)
	R‐TGGATATAAGTTCCTTCGC			
*lukED*	F‐CCATCGTTTATCACTACACTAT	35 cycles (95°C ‐ 30 s; 55°C ‐ 30 s; 72°C ‐ 45 s)	601	de Miranda et al. (2007)
	R‐CATAGTCAACAACATTTACAGC			
*lukPVL*	F‐ATCATTAGGTAAAATGTCTGGACATGATCCA	35 cycles (95°C ‐ 30 s; 55°C ‐ 30 s; 72°C ‐ 45 s)	433	Jarraud et al. (2002)
	R‐GCATCAAGTGTATTGGATAGCAAAAGC			
*ermA*	F‐AAGCGGTAAACCCCTCTGA	35 cycles (95°C ‐ 30 s; 55°C ‐ 30 s; 72°C ‐ 30 s)	190	Strommenger et al. (2003)
	R‐ TTCGCAAATCCCTTCTCAAC			
*ermB*	F‐ CTATCTGATTGTTGAAGAAGGATT	35 cycles (95°C ‐ 30 s; 55°C ‐ 30 s; 72°C ‐ 30 s)	142	Martineau et al. (2000)
	R‐GTTTACTCTTGGTTTAGGATGAAA			
*ermC*	F‐AATCGTCAATTCCTGCATGT	35 cycles (95°C ‐ 30 s; 55°C ‐ 30 s; 72°C ‐ 30 s)	299	Strommenger et al. (2003)
	R‐TAATCGTGGAATACGGGTTTG			
*ermT*	F‐ATTGGTTCAGGGAAAGGTCA	35 cycles (95°C ‐ 30 s; 55°C ‐ 30 s; 72°C ‐ 30 s)	536	Fessler et al. (2010)
	R‐GCTTGATAAAATTGGTTTTTGGA			
*aacA‐aphD*	F‐TAATCCAAGAGCAATAAGGGC	35 cycles (95°C ‐ 30 s; 55°C ‐ 30 s; 72°C ‐ 30 s)	227	Strommenger et al. (2003)
	R‐GCCACACTATCATAACCACTA			
*blaZ*	F‐ACTTCAACACCTGCTGCTTTC	35 cycles (95°C ‐ 30 s; 55°C ‐ 30 s; 72°C ‐ 30 s)	173	Martineau et al. (2000)
	R‐TGACCACTTTTATCAGCAACC			
*mecA*	F‐TCCAGATTACAACTTCACCAGG	35 cycles (95°C ‐ 30 s; 55°C ‐ 30 s; 72°C ‐ 45 s)	162	Oliveira and Lencastre (2002)
	R‐CCACTTCATATCTTGTAACG			
*ccrA2‐B*	F‐ATTGCCTTGATAATAGCCYTCT	30 cycles (95°C ‐ 30 s; 55°C ‐ 30 s; 72°C ‐ 45 s)	937	Boye et al. (2007)
	R‐TAAAGGCATCAATGCACAAACACT			
*ccrC*	F‐CGTCTATTACAAGATGTTAAGGATAAT		518	
	R‐CCTTTATAGACTGGATTATTCAAAATAT			
*IS1272*	F‐GCCACTCATAACATATGGAA		415	
	R‐CATCCGAGTGAAACCCAAA			
*mecA‐IS431*	F‐TATACCAAACCCGACAACTAC		359	
	R‐CGGCTACAGTGATAACATCC			

## Appendix 3 Variations in Single Nucleotide Polymorphisms (SNPs) Among the Genomes Analyzed

**Table:**

	SAMEA4520950	SAMEA4520956	SQ688	SAMEA4520951	SAMEA4520958	SAMN15902577	SAMEA4520957	SAMEA4520955	SAMN15214618	SQ696	SAMN15215142	SAMN15215159	SAMEA4520953	SAMEA4520952	SAMEA4520948	SAMN06847486	SAMN25285056	SAMN15215182	SAMEA4520949	SAMEA4520954	SAMEA4520959
SAMEA4520950	0	61	109	109	62	77	61	82	74	108	80	67	73	13	136	76	91	66	7	74	57
SAMEA4520956	61	0	94	91	5	32	0	64	55	93	61	48	55	60	117	31	46	47	54	56	10
SQ688	109	94	0	140	95	110	94	113	71	27	77	64	104	112	163	109	124	63	106	105	90
SAMEA4520951	109	91	140	0	92	107	91	67	104	139	110	97	58	109	166	106	121	96	103	57	87
SAMEA4520958	62	5	95	92	0	33	5	65	56	94	62	49	56	61	118	32	47	48	55	57	11
SAMN15902577	77	32	110	107	33	0	32	80	71	109	77	64	71	76	133	1	62	63	70	72	28
SAMEA4520957	61	0	94	91	5	32	0	64	55	93	61	48	55	60	117	31	46	47	54	56	10
SAMEA4520955	82	64	113	67	65	80	64	0	77	112	83	70	31	82	139	79	94	69	76	32	60
SAMN15214618	74	55	71	104	56	71	55	77	0	70	26	25	68	73	130	70	85	24	67	69	51
SQ696	108	93	27	139	94	109	93	112	70	0	76	63	103	111	162	108	123	62	105	104	89
SAMN15215142	80	61	77	110	62	77	61	83	26	76	0	31	74	79	136	76	91	30	73	75	57
SAMN15215159	67	48	64	97	49	64	48	70	25	63	31	0	61	66	123	63	78	1	60	62	44
SAMEA4520953	73	55	104	58	56	71	55	31	68	103	74	61	0	73	130	70	85	60	67	23	51
SAMEA4520952	13	60	112	109	61	76	60	82	73	111	79	66	73	0	135	75	90	65	6	74	56
SAMEA4520948	136	117	163	166	118	133	117	139	130	162	136	123	130	135	0	132	143	122	129	131	113
SAMN06847486	76	31	109	106	32	1	31	79	70	108	76	63	70	75	132	0	61	62	69	71	27
SAMN25285056	91	46	124	121	47	62	46	94	85	123	91	78	85	90	143	61	0	77	84	86	42
SAMN15215182	66	47	63	96	48	63	47	69	24	62	30	1	60	65	122	62	77	0	59	61	43
SAMEA4520949	7	54	106	103	55	70	54	76	67	105	73	60	67	6	129	69	84	59	0	68	50
SAMEA4520954	74	56	105	57	57	72	56	32	69	104	75	62	23	74	131	71	86	61	68	0	52
SAMEA4520959	57	10	90	87	11	28	10	60	51	89	57	44	51	56	113	27	42	43	50	52	0
